# Impact of Chronic Stress Protocols in Learning and Memory in Rodents: Systematic Review and Meta-Analysis

**DOI:** 10.1371/journal.pone.0163245

**Published:** 2016-09-23

**Authors:** Pedro Silva Moreira, Pedro R Almeida, Hugo Leite-Almeida, Nuno Sousa, Patrício Costa

**Affiliations:** 1 Life and Health Sciences Research Institute (ICVS), School of Health Sciences, University of Minho, Braga, Portugal; 2 ICVS/3B’s, PT Government Associate Laboratory, Braga/Guimarães, Portugal; 3 Clinical Academic Center–Braga, Braga, Portugal; 4 School of Criminology, Faculty of Law, University of Porto, Porto, Portugal; Indian Institute of Toxicology Research, INDIA

## Abstract

The idea that maladaptive stress impairs cognitive function has been a cornerstone of decades in basic and clinical research. However, disparate findings have reinforced the need to aggregate results from multiple sources in order to confirm the validity of such statement. In this work, a systematic review and meta-analyses were performed to aggregate results from rodent studies investigating the impact of chronic stress on learning and memory. Results obtained from the included studies revealed a significant effect of stress on global cognitive performance. In addition, stressed rodents presented worse consolidation of learned memories, although no significantly differences between groups at the acquisition phase were found. Despite the methodological heterogeneity across studies, these effects were independent of the type of stress, animals’ strains or age. However, our findings suggest that stress yields a more detrimental effect on spatial navigation tests’ performance. Surprisingly, the vast majority of the selected studies in this field did not report appropriate statistics and were excluded from the quantitative analysis. We have therefore purposed a set of guidelines termed PROBE (Preferred Reporting Orientations for Behavioral Experiments) to promote an adequate reporting of behavioral experiments.

## 1. Introduction

Stress exposure is associated with an activation of the hypothalamic-pituitary-adrenal (HPA) axis[1]. Although this relationship is thought to be bi-directional, here we focus on the causal effect of stress on HPA axis. Repeated stress exposure is known to lead to an excessive HPA axis activation, resulting in an overproduction of glucocorticoids (GCs). As a consequence, neurochemical and neuroanatomical alterations in several brain regions may be observed, including the hippocampus, prefrontal cortex, amygdala[2], dorsal striatum[3], nucleus accumbens[4], bed nucleus of the stria terminalis[5] and brain stem[6]. In the particular case of the hippocampus, a high density of GC receptors has been found[7–10]. Indeed, as a consequence of GCs overproduction, neuronal atrophy as well as decreased neurogenesis have been observed in the dentate gyrus of the hippocampal formation[11].

In experimental settings, several protocols of chronic stress induction have been devised. Among these, the Chronic Mild Stress (CMS) and the Chronic Restraint Stress (CRS) protocols have been the most widely used in behavioral research. In a typical CMS protocol, animals are exposed to unpredictable stressors over a varying period of time (from days to several weeks)[12]. On the contrary, in CRS protocols, the same stressor (restraining) is repeatedly applied[13, 14]. Some authors have demonstrated that the repeated exposition to stress leads to impaired hippocampal-dependent functions[15, 16] (also confront with[17, 18]) in several cognitive paradigms, such as the radial arm maze (RAM)[19], the Morris water maze (MWM)[20], the novel object recognition task (NOR)[21], and the Y Maze (YM)[22] (see also [23]^,^[24] for review). RAM and MWM are widely used experimental apparatus in which navigational and allocentric strategies are required; whereas, NOR and YM evaluate animals’ discrimination ability (novelty and path alternation, respectively).

The impact of chronic stress on cognitive performance is thought depend of biological (such as sex) and chronobiological (age) factors[25, 26]. Other aspects, including stress predictability, may also modulate these effects. For instance, it was reported that the implementation of predictable stressors enhances animals’ cognitive performance[27]. Adding further complexity to this issue, a recent study from our group revealed that the period of the day (diurnal/nocturnal) in which the stress protocol is implemented also modulates cognitive performance[28, 29].

This multi-factorial interplay may explain many of the inconsistencies found in the literature. Nevertheless, the deleterious impact of stress on cognitive functioning has been a cornerstone of decades of research. Many basic and clinical studies have departed from an assumption that has not always been confirmed. Therefore, it is critical to aggregate the data from multiple studies in order to clarify the abovementioned discrepancies. In this context, meta-analysis, though scarcely used in animal studies, is a powerful tool that incorporates the variability across studies, and allows the achievement of an overall estimate. Thus, it constitutes the most suitable means to untangle this issue. For this purpose, in this study we conducted a systematic review and meta-analyses in order to obtain an overall estimate of the impact of chronic stress on learning and memory in rodents. Furthermore, departing from our own observations, we also developed a set of guidelines with the aim of improving the quality of reporting of animal research experiments.

## 2. Materials and Methods

The systematic-review and meta-analyses adhered to PRISMA (Preferred Reporting Items for Systematic Reviews and Meta-Analyses[30]) guidelines, including search strategy, selection criteria, data extraction and data analysis (S1 File).

### 2.1. Literature search

A comprehensive literature search of electronic databases PubMed (http://www.pubmed.gov) and SCOPUS (http://www.scopus.com) was concluded in March 2014 with the following keywords: [‘learning’ AND ‘memory’] OR ‘morris water maze’ OR ‘novel object recognition’] AND [‘chronic’ AND ‘stress’] AND [‘mice’ OR ‘rats’]. Only experimental studies published in English were included in this analysis. Reviews, commentaries, as well as unpublished studies were not considered. Studies were selected if they met all the following criteria:

- implementation of a chronic stress protocol in post-weaning rodents, by applying CMS or CRS in one of the experimental groups;
- at least one control group was required;
- no other manipulation besides chronic stress was performed (e.g. drug treatments, enriched environment, physical exercise or others);
- experimental subjects were not genetically altered or had compromised functioning due to lesions or other manipulations;
- learning and memory were assessed in both control and experimental groups using validated tasks, such as the MWM, NOR, RAM and/or Y-M, after the implementation of the chronic stress protocol. Tasks requiring negative reinforcement, such as fear conditioning and passive avoidance tasks (see [31] and [32] for a review) were excluded. These tasks are characterized by an aversive and stressful nature. As a consequence, they were excluded with the aim of avoiding confounding effects.

### 2.2. Data extraction and management

Abstract selection: Two independent reviewers (PSM and PRA) selected eligible studies based on titles and abstracts’ screening. In the case of disagreements, a third reviewer (PC) decided if the study fulfilled the inclusion criteria.

To rule out subjectivity in the data gathering and entry process, data was independently extracted from eligible studies and recorded in separate databases by three reviewers (PC, PSM and PRA). Data from each study were abstracted using standardized forms in which the following characteristics were recorded: first author, publication year, stress protocol type, stress duration, sample size, animals’ age, gender and strain, and statistical measures for each behavioral parameter (means and standard deviations). Moreover, physiological indices (body weight, sucrose preference or corticosteroids’ levels) and behavioral measures (locomotor activity, anxious-like behavior) were also recorded.

If effect sizes could not be extracted/calculated from the available data, corresponding authors were asked (via e-mail contact) to provide additional statistical information. Afterwards, databases were compared and mismatching entries were identified and corrected upon discussion between the reviewers.

### 2.3. Data analysis

Heterogeneity was tested with the Cochran Q-test (p<0.10 indicates statistically significant heterogeneity[33]) and I^2^ statistic. I^2^ was calculated as I^2^ = [(Q–degree of freedom)/Q] ×100, where Q is the Cochran’s statistic. I^2^ values of 25, 50 and 75 represent low, medium and high heterogeneity, respectively. If high and significant heterogeneity (I^2^>75) was detected, a random-effects model (the Restricted Maximum-Likelihood method) was used to calculate the summary of pooled prevalence estimates. Otherwise, a fixed-effects model (the Mantel-Haenszel method) was preferred.

The presence of potential publication bias was examined through the visual inspection of funnel plot asymmetry, and statistically tested using the rank correlation method from Begg and Mazumdar (p<0.05 represents statistically significant publication bias).

Statistical analysis was conducted using Metafor package[34] in R software.

## 3. Results

### 3.1. Study selection

**Fig 1:**
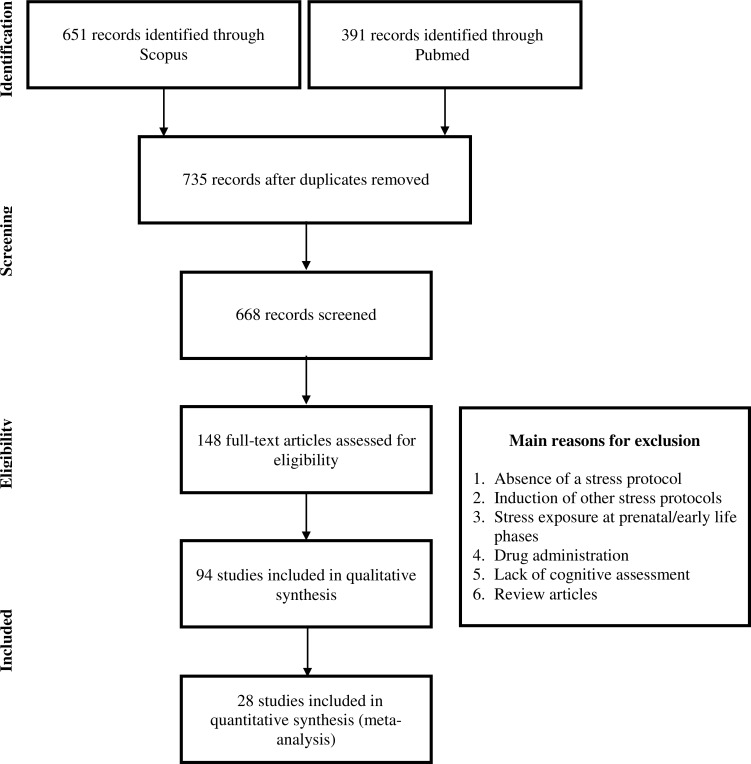
PRISMA diagram. Different phases of studies’ selection for conducting qualitative and quantitative analyses.

PRISMA diagram (Fig 1) illustrates the process of study selection.

The initial search yielded 1042 results, 335 of these were duplicated. Sixty-seven studies were excluded due to (1) not being written in English or (2) consisting of non-original research studies (*e*.*g*. reviews). During the initial screening (title and abstract), the main reasons for exclusion were: (1) absence/no implementation of stress protocol; (2) induction of stress (acute or chronic) protocols not considered in this work; (3) stress exposure at prenatal/early life phases; (4) stress implemented as a consequence of drug administration (*e*.*g*. dexamethasone); and (5) lack of cognitive assessment. At the end of the initial screening, 148 studies were selected for full-text review. During full-text review, 54 studies were further excluded, mainly due to: (1) absence of learning and memory assessment with validated behavioral tasks; (2) implementation of stress protocols not considered in the current work or absence of implementation of a chronic stress model; (3) use of fear conditioning protocols; (4) non-original research (review articles); (5) absence of control group.

Afterwards, for the selected studies, statistical measures were obtained either from the published paper (n = 15) or through e-mail contact to the corresponding authors (n = 13). Measures for the remaining studies could not be obtained due to lack of response of corresponding authors to email contacts. Thus, experiments in which the necessary measures for conducting the meta-analysis could not be calculated (n = 66), were included only in the systematic review. For the remaining, Cohen’s *d* (and the associated variance) was calculated as a measure of effect size (and the deviation measure). Cohen’s Kappa revealed a fair/good agreement between raters in the selection of the studies for the systematic review and meta-analyses (Kappa = .413, *p* < .001[35]).

### 3.2. Systematic review findings

Among the selected studies, chronic restraint (n = 49) and chronic mild (n = 45) stress protocols were implemented. With respect to learning and memory assessment, MWM was used in 50 experiments, NOR in 16, RAM in eight, and Y-Maze in 14. In five studies, other tasks [Hebb-Williams maze and labyrinth food finding test (n = 2); T-maze (n = 3)] were used. For studies performing more than one task, the results were analyzed individually. The majority of studies included only male animals, with only seven studies reporting findings from female rodents. With respect to animals’ strain, 38 studies used Wistar rats, 32 male Sprague-Dawley and four Long-Evans. In mice studies, seven used C57BL mice, five Kunming mice, four ICR mice, two BALB/C mice, one Laca mice and one study used albino Swiss mice. With respect to physiological changes following stress implementation, the stress group displayed reduction in body weight in 82% of the studies, stressed animals presented decreased sucrose preference in 92% of the studies, and corticosterone levels were augmented in stressed animals in 83% of the studies.

#### 3.2.1. Morris water maze

Overall, studies revealed that different types of chronic stress produce changes in cognition. Specifically, with respect to the MWM task, the majority of the studies (n = 28) report an absence of significant differences between the latency to reach the platform in the first acquisition day (Table 1). On the other hand, differences seemed to arise in the second, and especially third acquisition day, in which stressed animals displayed significantly more latency to find the hidden platform. Swimming speed was reported in only 12 studies. Of these, no differences were reported in eight studies, lower speed on stressed animals were found in one study, higher swimming speed on stressed animals was reported in three studies.

**Table 1:** Description of the studies using the MWM task. Characteristics: type of stress, duration, sex, age (and/or initial body weight), comparison of performance on acquisition days, probe and reversal parameters and differences in corticosterone (or sucrose preference) and body weight between stressed and control animals.

Study ID	Type of Stress	Animals	Sex	Age/	Acquisition^a^	Probe^a^	Reversal^a^	Stress Duration	COR/	BW	MA
				BW					SPT		
Bessa et al, 2009[44]	CMS	Wistar rats	Male	300–400g	Lat: —	—	S ↓ TQ	6 weeks	S ↓ SP	—	Yes
				3M	Speed: —		S ↑ OQ				
					Dist: S = C						
de Vasconcellos et al, 2005[45]		Wistar rats	Male	60d	Lat: —	S ↓ X	—	40 days	S ↑ COR	—	Yes
					Speed: —	S ↓ TQ					
					Dist: —	S ↑ OQ					
						S = C lat					
First et al, 2011[46]		Wistar rats	Male	8w	Lat: S = C	—	—	5 weeks	—	S = C	No
					Speed: —						
					Dist: —						
Kasar et al, 2009[47]		Wistar rats	Male	250–300g	Lat: S ↑ (day 3)	S = C	—	21 days	—	—	Yes
					Speed: —			6h/day			
					Dist: —						
Quan etal, 2011[48]		Wistar rats	Male	180–220g	Lat: S ↑ (1st session)	S = C X	S **↓** X	21 days	S ↓ SP	S ↓	No
					Speed: —	S = C TQ	S ↓ TQ				
					Dist: —		S ↑ OQ				
Sandi et al, 2006[49]		Wistar rats	Male	12M	Lat: S ↑ (1–3d)	—	—	4 weeks	S ↑ COR	S ↓	Yes
					Speed: S ↑ (day 1)						
					Dist: —						
Sun et al, 2006[50]		Wistar rats	Male	150–200g	Lat: S ↑ (2–3d)	—	—	6 weeks	—	—	Yes
					Speed: —						
					Dist: —						
Tagliari et al, 2011[51]		Wistar rats	Male	60d	Lat: S ↑ (4–6d)	S ↑ lat	—	40 days	—	—	No
					Speed: —	S ↓ TQ					
					Dist: —	S ↑ OQ					
Touyarot et al, 2004[52]		Wistar rats	Male	4M	Lat: S = C (1–3d)	S = C	—	21 days	S = C COR	—	No
					Speed: S = C						
					Dist: —						
Touyarot et al, 2004[52]		Wistar rats	Male	4M	Lat: S ↑ (2d)	S ↓ TQ	—	21 days	S = C COR	—	No
					Speed: S = C						
					Dist: —						
Cunningham et al, 2009[53]		Sprague-Dawley rats	Male	175–200g	Lat: S = C	—	—	10 days	—	—	No
					Speed: —						
					Dist: S = C						
First et al, 2013[54]		Sprague-Dawley rats	Male	8w/300g	Lat: S = C	—	—	4 weeks	S: ↓ SP	S ↓	No
					Speed: —						
					Dist: —						
Gouirand et al, 2005[55]		Sprague-Dawley rats	Male	45–60d	Lat: S ↓ (2–3d)	S ↓ dist	—	10 days	S = C COR	—	Yes
					Speed: —	S ↓ speed					
					Dist: —	S = C X					
						S = C TQ					
Isgor et al, 2004[56]		Sprague-Dawley rats	Male	21d	Lat: S ↓ (trial 1)	S = C	—	28 days	S ↑ COR	S ↓14%	No
					Speed: —						
					Dist: —						
Isgor et al, 2004[56]		Sprague-Dawley rats	Male	21d	Lat: S ↓ (trial 1)	S = C	—	28 days	S ↑ COR	S = C	No
					Speed: —						
					Dist: —						
Kallarackal et al, 2013[57]		Sprague-Dawley rats	Male	3–4w	Lat: —	—	—	6–7 weeks	S: ↓ SP	—	No
					Speed: S = C						
					Dist: —						
Li et al, 2009[58]		Sprague-Dawley rats	Male	150–180g	Lat: S ↑ (1–3d), S = C (6–8d)	S ↓ X	—	21 days	S: ↓ SP 23%	S = C	Yes
					Speed: S = C	S ↓ dist					
					Dist: —	S ↓ speed					
						S ↓ TQ					
Xi et al, 2011[59]		Sprague-Dawley rats	Male	200g	Lat: S ↑ (2–4d)	S ↓ X	—	42 days	S: ↓ SP	—	Yes
					Speed: —	S ↓ TQ					
					Dist: —	S = C Speed					
Zheng et al, 2006[60]		Sprague-Dawley rats	Male	150–200g	Lat: S ↑ (4–6d)	S ↓ X	—	4 weeks	S: ↓ SP	S ↓	No
					Speed: —						
					Dist: —						
Hill et al, 2005[61]		Long-Evans rats	Male	70d/300g	Lat: S = C	—	S ↑ lat	21 days	S ↑ COR 500%	—	No
					Speed: —		S ↑ OQ				
					Dist: —		S = C Speed				
Rinwa & Kumar, 2014[15]		Laca mice	Male	12w	Lat: S ↑ (1–3d)	S ↓ TQ	—	4 weeks	S ↑ COR	—	No
				20–30g	Speed: S ↑						
					Dist: —						
Bian et al, 2012[62]		Kunming mice	Male	3–4M	Lat: S ↑ (first 5d)	S ↓ TQ	—	40 days	—	—	No
				35–40g	Speed: —						
					Dist: —						
Liao et al, 2013[63]		Kunming mice	Male	30–35g	Lat: S ↑	S ↓ TQ	—	4 weeks	—	S = C	Yes
					Speed: —	S ↑ OQ					
					Dist: —						
Zhang et al, 2007[64]		Kunming mice	Male	20±2g	Lat: S ↑	—	—	21days	—	—	Yes
					Speed: —						
					Dist: S ↓						
Song et al, 2006[65]		ICR mice	Male	30–35g	Lat: S ↑ (first 5 days)	S ↓ TQ	—	4 weeks	S ↑ COR	S ↓	Yes
				7w	Speed: S = C (first 5 days)						
					Dist: —						
Bisaz et al, 2011[66]		C57BL/6J mice	Male	3M	Lat: —	S ↑ speed	S = C	4 weeks	S ↑ COR	S ↓	Yes
					Speed: S ↑ (first 3 days)	S ↑ dist					
					Dist: S ↑ (day 3)						
Cuadrado-Tejedro et al, 2011[67]		C57BL/6J mice	Female	8M	Lat: S ↑	S ↓ TQ	—	6 weeks	—	—	No
					Speed: S = C						
					Dist: —						
Liu et al, 2010[68]		BALB/c mice	Male	8w	Lat: S ↑ (3–6d)	—	—	4 weeks	S: ↓ SP 60%	—	Yes
					Speed: —						
					Dist: —						
Abidin et al, 2004[69]	CRS	Wistar rats	Male	3M	Lat: S ↑ (3–6d)	S ↓ TQ	—	21 days	S ↑ COR 600%	—	Yes
				300–350g	Speed: —	S ↑ OQ					
					Dist: —						
Ghadrdoost et al, 2011[16]		Wistar rats	Male	220±10g	Lat: S ↑ (4–5d)	S ↑ lat	—	21 days	S ↑ COR	—	No
					Speed: —	S ↓ TQ		6h/day			
					Dist: —						
Kitraki et al, 2004[70]		Wistar rats	Male and Female	—	Lat: S ↑	S ↑ OQ	—	21 days	S ↓ COR males	—	No
					Speed: —			6h/day	S ↑ COR females		
					Dist: —						
Kumar et al, 2009[71]		Wistar rats	Male	3M	Lat: S ↑ (2–6d)	S ↑ lat	—	21 days	—	—	No
					Speed: —	S ↓ TQ		6h/day			
					Dist: —						
Sandi et al, 2003[72]		Wistar rats	Male	150–175g	Lat: —	—	S = C	21 days	—	—	Yes
					Speed: —			6h/day			
					Dist: S ↑ (day 5)						
Trofimiuk & Braszko, 2013[73]		Wistar rats	Male	6w	Lat: S ↑ (2–3d)	—	—	21 days	—	—	Yes
					Speed: —			2h/day			
					Dist: —						
					S ↑ time in border						
Walesiuk & Braszko, 2009[17]		Wistar rats	Male	300–350g	Lat: S = C	—	—	21 days	—	—	No
					Speed: —			2h/day			
					Dist: —						
Walesiuk et al, 2005[74]		Wistar rats	Male	150–160g	Lat: S = C	S = C TQ	—	21 days	—	—	No
					Speed: —			2h/day			
					Dist: —						
Wattanathorn et al, 2013[75]		Wistar rats	Male	180–220g	Lat:	—	—	21 days	—	—	No
					Speed: —			2h/day			
					Dist: —						
Green & McCormick, 2013[13]		Sprague-Dawley rats	Male	22D	Lat:	S = C TQ	—	15 days	—	—	No
					Speed: —			1h/day			
					Dist: S = C						
Meng et al, 2013[76]		Sprague-Dawley rats	Male	227.2 ± 3.6g	Lat: S = C	S ↓ TQ	—	21 days	—	S ↓	No
					Speed: —	S = C X		3h/day			
					Dist: —						
Wang et al, 2009[77]		Sprague-Dawley rats	Male	8w	Lat: S = C (5d)	—	—	14 days	—	S ↓	No
					Speed: S = C			6h/day			
					Dist: —						
Wright & Conrad, 2008[78]		Sprague-Dawley rats	Male	300g	Lat: S = C (1–3d)	—	—	21 days	—	S ↓	No
					Speed: —			6h/day			
					Dist: S = C						
Wright & Conrad, 2008[78]		Sprague-Dawley rats	Male	300g	Lat: S ↑, S = C (2–3d)	—	—	21 days	—	S ↓	No
					Speed: —			6h/day			
					Dist: S ↑ (day 1)						
Xu et al, 2009[79]		Sprague-Dawley rats	Male	230–250g	Lat: S ↑ (first 5 days)	S ↑ lat	—	21 days	S ↑ COR	—	No
					Speed: S = C			6h/day			
					Dist: —						
Radecki et al, 2005[80]		Long-Evans rats	Male	397–405g	Lat: S ↑ (day 3)	S ↓ TQ	—	7 days	S ↑ COR 300%	—	No
					Speed: —			2h/day			
					Dist: —						
Liu et al, 2013[81]		Kunming mice	Male	18–22g	Lat: S ↑ (2–3d)	S ↓ TQ	—	21 days	S ↑ COR	—	No
					Speed: —			6h/day			
					Dist: —						
Tian et al, 2013[82]		Kunming mice	Male	10–12w	Lat: S ↑ (3–5d)	S ↓ TQ	—	21 days	S ↑ COR	—	No
					Speed: —			8h/day			
					Dist: —						
Muto et al, 2010[83]		ICR mice	Male	6w	Lat: S ↑ (day 5)	S = C TQ	—	4 weeks	—	S ↓	No
				30–32g	Speed: —			8h/day			
					Dist: —						
Nagata et al, 2009[14]		ICR mice		7w	Lat: S ↑ (2–6d)	S ↓ TQ	—	4 weeks	—	S ↓	No
					Speed: —			10h/day			
					Dist: —						
Delgado-Morales et al, 2012[84]		C57BL/6J mice	Male	—	Lat: S = C	S = C TQ	—	9 days	S ↑ COR	—	No
					Speed: —	S = C OQ		2h/day			
					Dist: —						
Pawlak et al, 2005[85]		C57BL/6J mice		8–12w	Lat:	S = C TQ	—	3 weeks	—	—	No
					Speed: —			6h/day			
					Dist: —						

CRS–Chronic Restraint Stress; CMS–Chronic Mild Stress; BW–initial body weight (when age is not referred); S–stress group; lat–latency; dist–total distance; ND–no differences; TQ–time spent in target quadrant; OQ–time spent in opposite/old quadrant; X–number of crossings; MA–included in the quantitative analysis

^a^Stress in comparison with control group.

Results relative to the probe test were reported in 33 studies. Of these, nine reported no differences between groups in the assessed parameters (time spent and number of crossings over the target quadrant and overall swimming speed). Four studies reported less crossings over the target in the stress group while one study reported the opposite pattern. Seventeen studies reported that stressed animals spent significantly less time in the target quadrant, only one study reported more time spent in the target quadrant by stressed animals, and no differences were found in the remaining seven studies. Swimming speed during the probe trial was reported in four studies with mixed results being reported: stresses animals displayed slower speed in two studies, higher speed in one, and no differences to the control group in stressed animals.

Regarding animals’ performance on the reversal test, stressed animals were found to spent more time in the original quadrant in three studies and fewer crossings in one. With respect to the total time spent in target quadrant, results were less consensual between reports: two studies indicated that stressed rodents spent less time in the target quadrant, and two studies revealed an absence of differences in this parameter.

#### 3.2.2. Novel object recognition

The performance of control and stressed animals on the NOR task was assessed in 11 studies (Table 2). The majority of studies reported an absence of differences in the total time exploring new objects between stressed and control animals. Only one study revealed a reduced total exploration time in stressed animals. On the other hand, five studies revealed that stressed animals display reduced exploration of new objects (as measured by the difference between novel and familiar objects). Two studies reported no differences between the groups. With respect to the discrimination index (DI, calculated as the difference between time spent exploring novel and familiar objects [36]), five studies reported a significantly reduced DI in stressed animals, while two studies reported no differences between the groups.

**Table 2:** Description of the studies using the NOR task. Characteristics: type of stress, duration sex, age (and/or body weight) and comparison of scores on object recognition and discrimination index between stressed and control animals.

Study ID	Type of Stress	Animals	Sex	Age/BW	Object recognition	Discrimination Index^a^	Stress Duration	COR/SPT	Body Weight	MA
Briones et al, 2012[86]	CMS	Wistar rats	Male	180–200g	S = C	—	6 weeks	S ↓ SP	S ↓	No
Llorent et al, 2011[87]		Wistar albino rats	Male and Female	—	—	S ↓	15 days			No
Parihar et al, 2011[27]		Sprague-Dawley rats	—	3M	—	S ↑	28 days	—	—	No
							2h/day			
Elizalde et al, 2008[88]		C57BL/6J mice	Male	8–10w	—	S ↓	6 weeks	S ↓ SP	S = C	No
Solas et al, 2013[89]		C57BL/6J mice	—	3M/24M	—	S ↓	6 weeks	S ↑ COR	—	Yes
Balk et al, 2011[90]	CRS	Wistar rats	Male	60D	S ↓	—	40 days	—	—	No
							1h/day			
Braszko et al, 2013[91]		Wistar rats	Male	2M	S ↓	S = C	21 days	—	—	Yes
							2h/day			
Trofimiuk & Braszko, 2013[73]		Wistar rats	Male	6w	S ↓	S = C	21 days	—	—	Yes
							2h/day			
Trofimiuk et al, 2014[92]		Wistar rats	Male	6w	S ↓	S = C	21 days	—	—	Yes
							2h/day			
Waleziuk et al, 2005[74]		Wistar rats	Male	150–160g	S ↓	S ↓	21 days	—	—	No
							2h/day			
Abush & Akirav, 2013[93]		Sprague-Dawley rats	Male	45d	S ↓	—	14 days	ND SP	S ↓	Yes
				200g			1h/day			
Bowman & Kelly, 2012[94]		Sprague-Dawley rats	Female	8w	S = C	—	35 days	—	S ↓	No
							6h/day			
Gomez et al, 2013[95]		Sprague-Dawley rats	Male	3M	S = C	—	7 days	S ↑ COR	S ↓	No
				220g			6h/day			
Gomez et al, 2013[95]		Sprague-Dawley rats	Male	3M	S = C	—	7 days	—	S ↓	No
				220g			6h/day			
Meng et al, 2013[76]		Sprague-Dawley rats	Male	227.2±3.6g	—	—	21 days	—	S ↓	No
							3h/day			
Nagata et al, 2009[14]		ICR mice	—	7w	—	S ↓	6 weeks	—	S ↓	No
							10h/day			

CRS–Chronic Restraint Stress; CMS–Chronic Mild Stress; S–stress group; ND–no data; BW–initial body weight (when age is not referred); MA–included in the quantitative analysis

^a^stress group in comparison to control group.

#### 3.2.3. Y-Maze

Among the selected studies, 14 experiments were conducted with the YM (Table 3). Of these, two studies did not report comparisons between groups. In the remaining studies, cognitive performance was assessed based on the number of entries in novel arms. No differences between groups were observed in six studies; in four studies, stressed animals had less entries; and in four studies, stressed animals over-performed control animals in four studies.

**Table 3:** Description of the studies using the RAM task. Characteristics: type of stress, duration, sex, age (and/or body weight) and comparison of learning performance between stressed and control animals.

Study ID	Type of Stress	Animals	Sex	Age/BW	RAM-Learning^a^	Stress Duration	COR/SPT	Body Weight	MA
Noorafshan et al, 2013[96]	CMS	Sprague-Dawley rats	Male	260±20g	S ↓ CR	56 days	S ↓ SP 40%	—	Yes
					S ↑ errors				
Srikumar et al, 2006[97]	CRS	Wistar rats	Male	200–250g	S ↓ CR	21 days	—	—	No
				2–2.5M		6h/day			
Veena et al, 2009[98]	CRS	Wistar rats	Male	2–2.5M	S ↓ CR	21 days	—	—	No
				200–220g		6h/day			
Waleziuk et al, 2009[17]	CRS	Wistar rats	Male	300–350g	S ↑ errors	21 days	—	—	No
						2h/day			
Bowman et al, 2003[18]	CRS	Sprague-Dawley rats	Female	55–60D	S ↑ CR	21 days	S ↑ COR	S ↓	No
						1h/day			
Bowman et al, 2003[18]	CRS	Sprague-Dawley rats	Female	55–60D	S = C	28 days	S ↑ COR	S = C	No
						1h/day			
Hutchinson et al, 2012[99]	CRS	Sprague-Dawley rats	Male	275g	S = C	21 days	—	S ↓	No
						6h/day			
Mika et al, 2012[100]	CRS	Sprague-Dawley rats	Male	250–275g	S ↑ errors	28 days	—	S = C	No
						6h/day			

CRS–Chronic Restraint Stress; CMS–Chronic Mild Stress; S–stress group; BW–initial body weight (when age is not referred); CR–correct responses; MA–included in the quantitative analysis

^a^stress group in comparison to control group.

#### 3.2.4. Radial arm maze

In the eight studies where the RAM task was used (Table 4), an obvious effect of stress on cognitive performance was noted: with the exception of two studies, stressed animals displayed more errors and less correct choices.

**Table 4:** Description of the studies using the Y-M task. Characteristics: type of stress, duration, sex, age (and/or body weight) and comparison of performance between stressed and control animals.

Study ID	Type of Stress	Animals	Sex	Age/BW	Y-M^a^	Stress Duration	COR/	BW	MA
							SPT		
Henningsen et al, 2009[101]	CMS	Wistar rats	Male	230±10g	S ↑ DI	7 weeks	S ↓ SP 40%	S = C	No
Palumbo et al, 2010[102]		c57bl mice	—	2M	S = C	6 weeks	S ↑ COR	—	Yes
Palumbo et al, 2010[102]		Balbc mice	—	2M	S ↓ DI	6 weeks	S ↑ COR	—	Yes
Bellani et al, 2006[103]	CRS	Sprague-Dawley rats	Male	35D	S = C	21 days	S ↑ COR	S ↓	No
						6h/day			
Conrad et al, 1996[22]		Sprague-dawley rats	Male	200–250gm	S ↑ DI	21 days	—	—	No
						6h/day			
Conrad et al, 2003[104]		Sprague-Dawley rats	Male and Female	—	S ↑ DI	21 days	—	S ↓	No
						6h/day			
Gomez et al, 2013[95]		Sprague-Dawley rats	Male	3M	S ↓ DI	7 days	S ↑ COR	S ↓	No
				220g		6h/day			
McLaughlin et al, 2007[105]		Sprague-Dawley rats	Male	—	S = C	21 days	—	S ↓	No
						6h/day			
McLaughlin et al, 2007[105]		Sprague-Dawley rats	Male	—	S = C	10 days	—	S ↓	No
						6h/day			
Wright & Conrad, 2005[106]		Sprague-Dawley rats	Male	300–400g	S = C	21 days	—	S ↓	No
						6h/day			
Wright & Conrad, 2008[78]		Sprague-Dawley rats	Male	300g	S ↓ DI	21 days	—	S ↓	No
						6h/day			
Wright et al, 2006[39]		Sprague-Dawley rats	Male	225–250g	S ↑ DI	21 days	S = C	S ↓	No
						6h/day			
Kleen et al, 2006[107]		Sprague-Dawley	Male	—	S ↓ DI	21 days	—	S ↓	No
						6h/day			
Chen et al, 2010[108]		ICR mice	Male	3w	S = C	28 days	—	S ↓	No
						6h/day			

CRS–Chronic Restraint Stress; CMS–Chronic Mild Stress; S–stress group; DI–Discrimination Index; BW–initial body weight (when age is not referred); ND–no data; MA–included in the quantitative analysis

^a^stress group in comparison to control group.

#### 3.2.5. Integration of findings

The comparison between control and stressed animals for the different tasks is summarized on Table 5. The test parameters that yielded the most significant differences between groups were the latency on the acquisition phase in the MWM test (Χ^2^_(2)_ = 23.3, p < .001), time spent in the target quadrant (Χ^2^_(2)_ = 17.2, p < .001) and the object recognition in the NOR test (p = .044). In addition, trends for statistical significance were also observed in the speed on the acquisition phase in the MWM test (p = .054) and in the time spent at the old quadrant (p = .054). Of note, stressed animals had reduced performance in all these parameters.

**Table 5:** Summary of groups’ comparisons on different MWM phases.

						
		Comparison between groups^a^				
Task	Parameter	Total	S = C	S↑	S↓	Sig^b^
MWM Acquisition						
	Latency	42	11 (26%)	28 (67%)	3 (7%)	Χ^2^_(2)_ = 23.3, p < .001
	Speed	12	8 (67%)	1 (8%)	3 (25%)	p = .054
	Distance	8	4 (50%)	1 (13%)	3 (38%)	p = .552
MWM Probe						
	Crossings	7	3 (43%)	0 (0%)	4 (57%)	p = .174
	Target quadrant	26	7 (27%)	1 (4%)	18 (69%)	Χ^2^_(2)_ = 17.2, p < .001
	Old quadrant	6	1 (17%)	5 (83%)	0 (0%)	p = .054
	Latency	5	1 (20%)	4 (80%)	0 (0%)	p = .136
	Speed	4	1 (25%)	1 (25%)	2 (50%)	p = .148
	Distance	3	0 (0%)	1 (33%)	2 (67%)	p = .111
MWM Reversal						
	Crossings	1	0 (0%)	0 (0%)	1 (100%)	p>.999
	Target quadrant	4	2 (50%)	0 (0%)	2 (50%)	p = .556
	Old quadrant	3	0 (0%)	3 (100%)	0 (0%)	p = .111
NOR						
	Object recognition	10	4 (40%)	0 (0%)	6 (60%)	p = .044
	Discrimination Index	9	3 (33%)	1 (11%)	5 (56%)	p = .319
Y-M						
	Discrimination Index	14	6 (43%)	4 (29%)	4 (29%)	p = .842
RAM						
	Correct responses	6	2 (33%)	1 (17%)	3 (50%)	p = .877
	Errors	3	0 (0%)	3 (100%)	0 (0%)	p = .111

^a^Number of studies in which each parameter was compared

^b^The differences between group prroportions were tested using chi-square statistics or multinomial test, depending on whether the assumptions for the chi-square statistic were verified or not, respectively

S–stress group.

### 3.3. Meta-analytic results

#### 3.3.1. Global analysis

**Fig 2:**
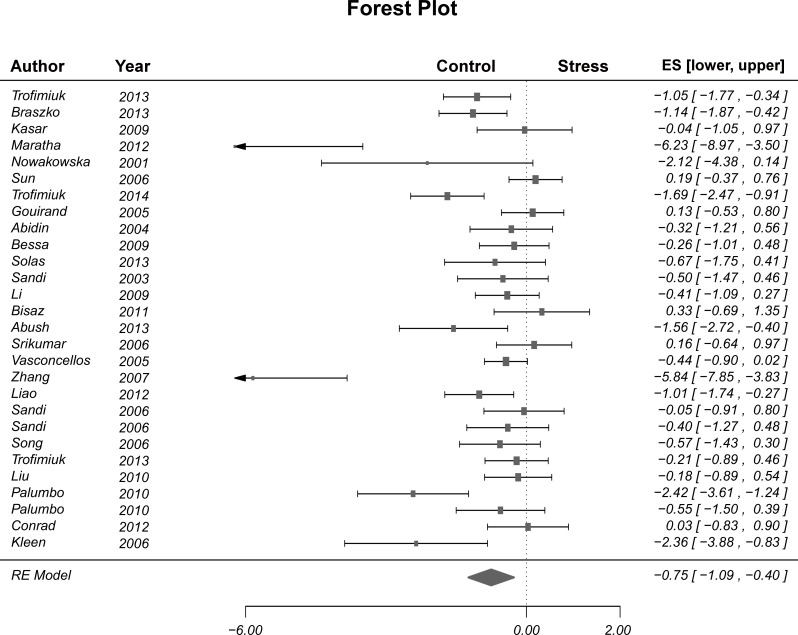
Forest plot. Overall effect of stress influence on general cognition. Individual lines represent each study included in the meta-analysis. The vertical dashed line represents absence of effects between stressed and control animals. The diamond located at the bottom of the figure represents the overall effect. As can be observed, the diamond does not cross the vertical line, which indicates that overall effect is significant.

A general cognitive parameter was calculated based on the scores on learning and memory measures assessed in the different tasks, by averaging the computed effect sizes for each reported measure. Significant heterogeneity among studies was verified (Q_(27)_ = 100.8, p < .001) and therefore a Random-Effects model was selected. An overall effect of stress on general cognition was noted (d = -0.75, SE = 0.18, p < .001), such that stressed animals present overall lower cognitive scores, compared with control animals. The overall effect is graphically represented on the Forest Plot (Fig 2). The test for funnel plot asymmetry revealed a significant result (t_(26)_ = -4.03, p < .001).

#### 3.3.2. Morris water maze

Omnibus analysis: The parameters assessed during the acquisition phase (latency, distance and swimming speed) and the probe trial (time spent in the target quadrant, number of crossings and swimming speed) were used to estimate a general cognitive effect size for each study. The total variability in the model was significantly affected by heterogeneity (Q_(15)_ = 40.5, p < .001). The Random-Effects model revealed a significant overall effect of stress on general MWM performance (d = -0.32, SE = 0.11, p < .001). A trend for a significant asymmetry was observed between studies included in this analysis (t_(14)_ = -2.11, p = .053).

Regarding the acquisition days on the MWM task, no significant differences were found in the latency to reach the platform on each of the first two days between groups: Day 1: d = 0.11, SE = 0.13, p = .399; Day 2: d = 0.13, SE = 0.13, p = .353. Regarding the third day, following the assessment of heterogeneity, (Q_(7)_ = 16.4, p = .022, accounting for 56.2% of the variability of the model), it was observed a significant overall effect of stress on the latency to reach the platform(d = 0.58, SE = 0.21, p = .007), with stressed animals presenting worse performance.

With respect to the probe trial, a marginally significant effect of heterogeneity was observed (Q_(5)_ = 10.6, p = .061), contributing for 53.7% (I^2^) of the variance in this model. Consequently, a Random-Effects model was preferred. A significant overall effect of stress was observed (d = -0.46, SE = 0.03, p = 0.029).

#### 3.3.3. Sensitivity analysis

Leave-one-out (sensitivity) analyses were conducted for each independent meta-analysis. It was observed that the exclusion of a single study did not yield significant changes in overall effects.

#### 3.3.4. Moderator and mediator effects

To account for the impact of stress implementation on the overall effect, moderator and mediator meta-analyses were conducted. Using random-effects categorical moderator models, it was observed that both CMS (d = -0.67, SE = 0.20, p = .001) and CRS (d = -0.83, SE = 0.28, p = .003) displayed significantly impact on learning and memory. In addition, restricted maximum-likelihood meta-regression analysis revealed that stress duration did not significantly affected the overall effect (R = -0.003, SE = 0.02, p = .841).

## 4. Discussion

### 4.1. General

In this work, we conducted a systematic review and meta-analytic procedures to study the effect of chronic stress on cognitive performance in mice and rats. Despite the observed heterogeneity, it can be generally concluded that the implementation of different protocols of chronic stress leads to alterations on cognitive functioning, particularly on the consolidation of learned memories. For instance, in accordance with a previously hypothesized biphasic effect of chronic stress on the central and peripheral nervous system, it could be expected that shorter exposure to stress would be beneficial to the organism, whereas longer stress exposures would lead to detrimental consequences[18]. Although this effect was apparent in the systematic review findings, the results obtained from the meta-regression failed to confirm a significant association between the duration of stress implementation and the overall estimates. Nevertheless, the absence of significant results may stem from the fact that in the majority of studies (64.3%) stress is implemented during 21 or 28 days, leading to a reduced variability between studies. As previously indicated, it is frequently accepted that the impact of chronic stress on cognitive function is dependent on GCs’ overproduction and concomitant hippocampal atrophy [37]. Yet, some studies included in this analysis report impairments on cognitive performance as a consequence of chronic stress co-occurring with normal levels of circulating corticosterone. It might be hypothesized that GCs exert a differential role on cognition, particularly on memory performance. In fact, it has been proposed that GCs have a dissociative impact on memory consolidation and retrieval [38]. Alternatively, it is also possible to hypothesize that the exposure to stress potentially affects cognitive performance, without affecting corticosterone levels. In line with this, it has been hypothesized that stress impairs cognition through a down-regulation of hippocampal glucocorticoid receptors’ levels and production of CA3 dendritic retraction[39]. It is also relevant to highlight that although in this work, we have focused on the effects of stress on HPA axis, this relationship is thought to be bi-directional. Indeed, HPA axis deregulation is known to contribute to the development of psychosomatic and psychiatric conditions, with its hyper-reactivity being itself associated to an inadequate response to stress[40].

### 4.2 Strengths and Limitations

Meta-analytic studies are characterized by high level of evidence, as they allow the computation of omnibus results from multiple studies, while accounting for the variability between individual works. Thus, one major contribution of this work relies on the estimation of overall effects. We expect that this work may serve as a rigorous means of estimating sample sizes, which will be critical for detecting true positive effects (*i*.*e*. to avoid type II errors). Simultaneously, this approach will also limit the maximum number of animals to use, which is in line with the Russell and Burch[41] recommendations expressed in the principles of 3Rs.

Nonetheless, results herein presented should be interpreted with some caution. The systematic review process is prone to criticism. On this, one can argue that the process of selecting studies may be itself biased, due to different factors such as the initial exclusion based only on abstracts’ reading or to the inclusion of studies from the same group of researchers. However, this was based on the widely recommended and most accepted practices for conducting systematic reviews. Another criticism may be related with the exclusion of tasks encompassing aversive learning. Several studies demonstrate that the implementation of stress conducts to an impaired performance in these tasks. Nevertheless, we decided to exclude these tasks with the goal of avoiding the influence of potential confounders.

Furthermore, a major concern raised in this work is related with the reduced number of studies included in the meta-analysis. This was particularly disappointing since a considerable number of studies met the inclusion criteria. However, most of the studies did not report appropriate statistics required for the computation of effect size measures. As a consequence, the meta-analytic calculations were estimated based on a reduced number of studies. This also precluded the appropriate control of covariates of interest, such as animals’ strain and age. As a good practice and following other research areas, research with animal models would benefit from a better data reporting. In particular, a comprehensive description of the appropriate statistics is of critical relevance, as it will allow an aggregation of results from different studies employing similar experimental manipulations. This aspect was also referred in a recent review that focused on the quality of experimental design in the field[42]. Another relevant issue highlighted in our work is related with the presence of publication bias. Although we were not able to test for publication bias in individual parameters due to a reduced number of studies reporting the same outcome, significant asymmetry was found on the global analyses.

In addition, aspects pertaining to the experiment organization, including lack of appropriate randomization or experimenter blinding, raise additional concerns. In particular, randomization was not reported in a considerable part of the studies and blindness was rarely referred. These factors highlight the relevance of improving experimental designs and the current guidelines in the reporting of the experiments with animals as a means to ensure an appropriate level of research evidence.

A further limitation is related with the reduced number of studies with female animals, which precluded the analysis of the moderating effect of sex. This would be of upmost importance, since it has been acknowledged that the effects of stress on anatomical, neuroendocrine and neurochemical variables and on cognitive performance measures varies between sexes[18, 43]. For instance, there is evidence showing that male rats, but not female, show impaired performance in the NOR task after 21 days of chronic stress. These results are also reflected at the neural and endocrine levels, where male rats show significant atrophy of apical dendritic branches of the CA3c pyramidal neurons. In contrast, female rats showed a decreased number of branch points in the basal dendritic area and revealed higher levels of plasma corticosterone both at baseline and during stress implementation[43]. These differences highlight the importance of characterizing the effects of stress, taking into account the sex of the animals. Moreover, based on our findings, it seems evident that more research with females should be undertaken, with the goal of better understanding the neurophysiological mechanisms, and protective factors, of cognitive decline following stress.

Finally, it was noted that structured procedures for the implementation of stress are still missing. As an example, CRS protocols varied between studies concerning duration of the restraint sessions (one to six hours) and extension of the protocol (from 14 to 28 days). Additionally, a recent study from our group demonstrated that methodological differences such as the implementation of stress protocol in the resting (light phase) or activity (dark phase) of the animal can differentially impact the performance on probe test[29]. Also, there is considerable heterogeneity with respect to behavioral assessments. For instance, there is high variability in the number of acquisition days to assess the animals’ performance on the MWM. Consequently, animals will have different training levels from experiment to experiment, which will likely induce alterations in the animals’ performance during the probe trial. Reported parameters are also exceedingly heterogeneous, with different measures being reported across studies, such as swimming speed, latency or distance to the hidden platform. Curiously, some authors reported an average of the assessed parameter during the acquisition days, while others presented these parameters during individual days/blocks/sessions. This also limits the assessment of animals’ learning curve throughout days.

### 4.3. PROBE–Preferred Reporting Orientations for Behavioral Experiments

In order to overcome the abovementioned limitations, herein we propose a guidance for reporting results in animal research, henceforth termed PROBE (Preferred Reporting Orientations for Behavioral Experiments) (Table 6). In this set of guidelines, we focus on distinct classes of factors that were experienced as crucial in the development of this work. Specifically, this guidance focus on several parameters, including: experimental conditions, biological factors, experimental organization (emphasizing both general aspects and those related to the implementation of stress protocols), experimental design and statistical analysis. As previously mentioned, the rationale behind the selection of these factors relies on our experience in the selection of studies to conduct this work. Overall, these guidelines are aimed to constitute a checklist to be progressively established in the animal research field in order to enhance the quality and accuracy of data reporting. We consider that this will allow an easier communication between different researchers and laboratories, by enabling the understanding of possible methodological differences that may lead to contrasting (and even contradicting) outcomes.

**Table 6:** PROBE–Preferred Reporting Orientations for Behavioral Experiments.

Class	Factors	Descriptors
**Experimental conditions**	Caging conditions	Cage type; number of individual/cage; bedding
	Diet	Diet type; regime (*e*.*g*. *ad libitum*)
	Environmental	Temperature/humidity, light cycle
	Experimental subjects’ provenience	Suppliers; in-house crossings
**Biological**	Species	—
	Strain	—
	Genotype	—
	Sex	—
	Age	Age in days, weeks or months
	Body weight	At several time points including pre- and post-experimental involvement
	Previous involvement in other experiments	—
**Experimental Organization (general)**	3Rs principle	Replacement, Reduction and Refinement
	Qualified researcher	—
	Experimental groups	Number of experimental groups. Detailed description of manipulations that were implemented
	Handling	duration, periodicity, procedures
	Subject Randomization	Were animals randomly distributed by groups; if not describe distribution criteria
	Blinding	Was the researcher who performed the behavioral assessment aware of animals' experimental group
	
**Experimental Organization (stress)**	Type of stress	Type of stress: Chronic Restraint Stress, Chronic Mild Stress, Early-life stress, Social stress
	Description of stressors	Description of the different stressors applied by day, if applicable
	Duration of stress	Number of days of chronic stress implementation
	Basal corticosterone levels after stress	Serum corticosterone levels after stress period in all experimental groups
	Assessment of anhedonic behavior	Assessment of anhedonia through the quantification of sucrose preference
	Assessment of anxiety-like behavior	Assessment of anxiety-like behavior by using a validated task such as the elevated plus maze
	Assessment of helplessnees behavior	Use of validated task (e.g. Forced Swimming Test/Tail suspension) to evaluate depressive-like behavior
	Interval between stress protocol and behavioral assessment	Time between the end of the implementation of the stress protocol and behavioral assessment
	Description of the behavioral assessment task	Task used for behavioral assessment (e.g. MWM, NOR, RAM, Y-M, Passive Avoidance)
	Duration of behavioral assessment	Number of trials in each stage (e.g. number of acquisition days, interval between acquisition and probe trial)
**Experimental design and statistical analysis**	A priori analysis	Confidence level (and consequently type I error), Statistical power (and consequently type II error) and sample size calculation
	Sample size	—
	Statistical measures of task parameters	Mean and standard deviations for each parameter assessed in behavioral tasks
	Effect size	Quantification of the magnitude of the effect of a given manipulation
	Excluded subjects and exclusion criteria	—

## 5. Conclusions

Cognitive dysfunction is a hallmark of chronic stress in humans. However, in rodents, divergent findings regarding the effects of chronic stress on cognitive performance have been reported. This raises serious concerns to the translation value of rodent models of chronic stress. Despite this heterogeneity, our meta-analytic work provides solid evidence that indeed rodents mimic this feature of human pathology. As a corollary of this work, we suggest a set of guidelines for adequate reporting of animal results. We expect this to be helpful in facilitating the aggregation of results in the field and potentiating an increased level of research evidence. Taken together, the present work may be a relevant reference for future studies, by potentiating a better research planning and reporting in work involving animal experimentation. This will also potentiate the validity (face, predictive and construct validities) of animal models and their translation value.

## Supporting Information

S1 File PRISMA Checklist. (DOC)

S2 File Database. (XLSX)
